# Hydraphiles: A Rigorously Studied Class of Synthetic Channel Compounds with *In Vivo* Activity

**DOI:** 10.1155/2013/803579

**Published:** 2013-01-15

**Authors:** Saeedeh Negin, Bryan A. Smith, Alexandra Unger, W. Matthew Leevy, George W. Gokel

**Affiliations:** ^1^Departments of Chemistry and Biochemistry and Biology, Center for Nanoscience, University of Missouri-St. Louis, St. Louis, MO 63121, USA; ^2^Department of Chemistry and Biochemistry, 236 Nieuwland Science Hall, University of Notre Dame, Notre Dame, IN 46556, USA; ^3^Notre Dame Integrated Imaging Facility, University of Notre Dame, Notre Dame, IN 46556, USA

## Abstract

Hydraphiles are a class of synthetic ion channels that now have a twenty-year history of analysis and success. In early studies, these compounds were rigorously validated in a wide range of *in vitro* assays including liposomal ion flow detected by NMR or ion-selective electrodes, as well as biophysical experiments in planar bilayers. During the past decade, biological activity was observed for these compounds including toxicity to bacteria, yeast, and mammalian cells due to stress caused by the disruption of ion homeostasis. The channel mechanism was verified in cells using membrane polarity sensitive dyes, as well as patch clamping studies. This body of work has provided a solid foundation with which hydraphiles have recently demonstrated acute biological toxicity in the muscle tissue of living mice, as measured by whole animal fluorescence imaging and histological studies. Here we review the critical structure-activity relationships in the hydraphile family of compounds and the *in vitro* and *in cellulo* experiments that have validated their channel behavior. This report culminates with a description of recently reported efforts in which these molecules have demonstrated activity in living mice.

## 1. Introduction

The outer membranes of organisms serve to enclose the functioning cell and separate it from the external environment [1]. This vital protective function is complicated by the need for the cell to permit the entry of nutrients and the egress of waste products. It is unclear what were the earliest barriers that permitted separate cells to evolve and ultimately to combine into higher life forms [2, 3]. Indeed, there is considerable scholarly effort currently underway in this area. What is clear is that along with the development of protective or confining membranes, structures and mechanisms had to evolve [4] that would permit selective passage of ions and molecules through them [5]. Today, the proteins that regulate ion balance and transmembrane transport are extremely complex molecules that interact directly with the membranes in which they are embedded, and they exhibit remarkable selectivity (specificity) in their chemical functions [6].

During the past two decades, considerable effort has been expended to develop synthetic amphiphiles that will insert into membranes and exhibit at least some of the functions of highly complex protein channels [7, 8]. There has been considerable success in this arena, and a number of reviews describe the efforts [9–13]. Our own effort in this area initially involved the compounds we have called “hydraphiles” [14]. These compounds are typically constructed from three crown ether macrocycles linked by spacer chains of varying lengths. They readily insert into bilayer membranes and conduct ions through them. When studied by planar bilayer conductance methods, they show the open-close behavior characteristic of protein channels [15]. These symmetrical molecules are nonrectifying and show toxicity to bacteria [16], yeast, and even to mammalian cells [17]. The toxic effect results from a rapid disruption of ion homeostasis [18]. 

The hydraphile molecules may be viewed as problematic in biological applications owing to their toxic effects. However, at concentrations below those at which they are toxic, they show no effect on bacterial growth, but exhibit a remarkable ability to enhance the efficacy of antibiotics [19]. Moreover, they can be used selectively in pathological conditions. An example is the recently reported use of hydraphiles in direct injection chemotherapy, as described below. 

## 2. Results and Discussion

### 2.1. The Design of a Synthetic Ion Channel

**Figure 1:**
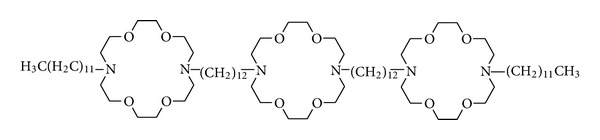
Structure of a hydraphile synthetic ion channel, **1**.

The initial design of the hydraphiles [20] in many ways paralleled contemporaneous channel designs developed by Jullien and Lehn [21] and by Carmichael et al. [22]. Our approach was based on biophysical data concerning protein channel properties and function because the X-ray structure of a channel protein was unavailable. Some guidance was taken from the solid state structure of bacteriorhodopsin, which is an integral membrane protein [23]. Estimates were made of the overall length that would be required for the molecule to function within a membrane. An important consideration was what element or module would serve as the amphiphile's head group and what, if anything, would serve as an energy-lowering element that would reside at the midplane of the bilayer. The various considerations and strategies were described several years ago in a focus article [24] and will be recounted here only in the general sense. The first structure to be prepared in this effort is shown in Figure 1. 

### 2.2. Design Variables, Strategies, and Syntheses

 The diaza-18-crown-6 macrocycle was chosen to function both as the amphiphilic head group and as what we call the “central relay.” Previous studies showed that diazacrowns could function effectively as amphiphile head groups [25, 26]. The 18-membered ring macrocycles were chosen because they are more readily prepared than other sizes of macrocycles of this type and because we expected them to bind K^+^ more strongly than Na^+^. In principle, this difference should confer Na^+^  >  K^+^ selectivity upon the channel because more strongly bound K^+^ would be released and transported less readily than weakly bound Na^+^. It was assumed that spacer chains within the bilayer would be extended. The distance spanned by each carbon-carbon bond in an extended gauche conformation is about 1.25 Å. Spacer chains of 12 carbons in length would therefore span about 15 Å. Two such chains, and the central macrocycle, would give an overall extension to the molecule of approximately 40 Å. The so-called insulator regime of a phospholipid bilayer is generally thought to have a thickness of about 30–35 Å.

The overall distance spanned by the medial macrocycle depends significantly upon whether it is parallel or perpendicular to the membrane's fatty acid chains. Initially, we surmised that the central macrocycle would be perpendicular to the fatty acid chains in the bilayer, but parallel to the two macrocyclic head groups. In this conformation, the macrocycle would span a distance closer to 30 Å than to 35 Å. However, it was thought that the central macrocycle would not need to be exactly parallel to the head group surfaces of the membrane; this would allow for some flexibility in the channel's overall length.

A key reason that the third, central, or medial macrocycle was included in the overall span of the channel was that chemical intuition suggested that some energy lowering element would be required at the midplane of the bilayer. The midplane is the region most remote from the polar head groups and therefore is the least polar portion of the head group-midplane-head group transmembrane gradient. The initial thought was that it would be energetically unfavorable for an ion to traverse a distance as large as 30–35 Å. An encounter between the ion and macrocycle might permit water exchange or a transient complexation interaction. It was further surmised that the transient contact would be energetically favorable. On mature consideration, it was apparent that any such complexation interaction between transporter and ion would require additional energy for release of the ion. Such interactions would restrict the flow of ions and therefore be unfavorable. Exchange of waters of solvation, however, was expected to be energetically favorable.

**Figure 2:**
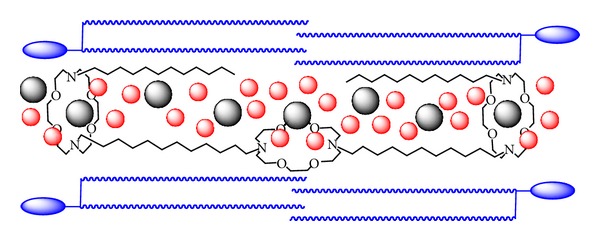
Schematic representation of hydraphile **1** positioned within the phospholipid bilayer.

The overall design required the “distal” macrocycles to function as head groups in the amphiphilic sense [27, 28] and as entry portals for ions. Hydrocarbon chains containing 12 carbons were used as covalent links. These dodecylene chains would connect the distal macrocycles with the medial macrocycle. Each distal crown was then attached to another 12-carbon chain in order to improve interactions with the fatty acid chains in the bilayer, to make the molecule more cylindrical, and to better shield the ion pathway. The chemical structure of the first synthetic hydraphile (**1**) is shown in Figure 1, and the biophysical evidence suggests that it is positioned within the bilayer membrane, in Figure 2. 

It is apparent from the structure of **1** that the first “hydraphile” is a symmetrical molecule. It contains three identical macrocyclic rings and four 12-carbon chains. One of the macrocycles is linked to two other macrocycles and two of the macrocycles are linked to dodecylene chains. The synthetic challenge was therefore to make a molecule that is overall symmetric and has several symmetrical elements, but in which both macrocycles, and dodecylene chains are connected in slightly different ways. This was accomplished by desymmetrizing the distal macrocycles and then linking them to the central macrocycle by alkylation or by acylation followed by reduction. 

### 2.3. Biophysical Experiments to Confirm Membrane Insertion and Channel Function

Characterization of the initial channel compound was done by using a dynamic NMR method developed by Riddell and coworkers [29]. In this technique, phospholipid liposomes (vesicles) are prepared in the presence of aqueous sodium chloride. The vesicles in the bulk suspension and the surrounding medium both contain sodium ions. Because the vesicles were prepared in the medium, the concentration of Na^+^ inside and outside of the vesicles is identical. A ^23^Na-NMR spectrum of this system shows a single resonance because Na^+^ ions within the liposomes and in the bulk phase experience the same buffer environment. If an NMR shift reagent such as Dy^3+^ is added to the external medium (the bulk aqueous phase), two ^23^Na resonances will be observed: those within the liposomes and protected from the influence of Dy^3+^ and those in the bulk phase. Because there is significantly more sodium in the external solution than is contained within the vesicles, the Dy^3+^-shifted resonances will be more intense and observed at different positions. If a compound that can insinuate itself into the membrane and lead to sodium exchange is added to the solution, ion transport through the membrane will be observed. This will lead to changes in the respective linewidths. From the change in linewidth, an ion exchange rate constant can be calculated [30].

**Figure 3:**
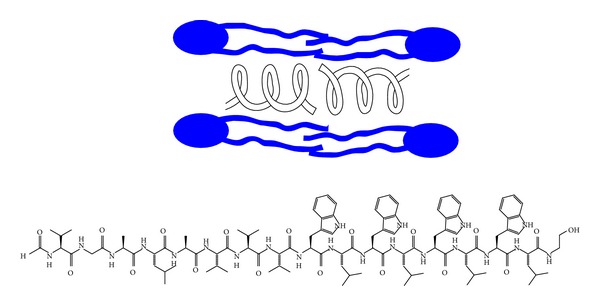
Structure of a gramicidin peptide. The generalized structure of several gramicidins is O=CH-L-X-Gly-L-Ala-D-Leu-L-Ala-D-Val-L-Val-D-Val-L-Trp-D-Leu-L-Y-D-Leu-L-Trp-D-Leu-L-Trp-NCH_2_CH_2_OH. Two gramicidin peptides coil as shown schematically to give an ion-conducting pore within the bilayer. X and Y indicate variability in the peptide sequence.

The experiment described, while valuable and reproducible, is both cumbersome and labor intensive. In our experiments, the synthetic channel compounds we prepared were always studied in parallel with the natural peptide channel former, gramicidin (Figure 3). Gramicidin is a natural bacterial peptide (mixture) that forms robust channels in bilayer membranes [31]. In our studies, whatever rate was observed with gramicidin, that rate was set arbitrarily to a value of 100. Whatever rates were observed for other channel compounds were then normalized to the gramicidin value. This permitted us to obtain reproducible rates at different times on different instruments when run by different operators. Compound **1**, shown in Figure 1, transported sodium cation through phospholipid bilayers at a rate equal to approximately 25–30% that of gramicidin. This may not seem impressive at first flush. Recall that excellent carriers such as valinomycin conduct about 10^4^ ions · sec^−1^ whereas pore formers typically conduct ions in the 10^6^–10^8^ ions · sec^−1^ range.

It should also be noted that a number of the synthetic ion channels were studied in a concentric tube apparatus intended to evaluate carrier transport [32]. All of the channel compounds studied transported ions across the bulk chlorocarbon membrane. There was, however, no correlation between the ability of these compounds to serve as carriers and their channel-like behavior compared to gramicidin's behavior in liposomal membranes.

**Figure 4:**
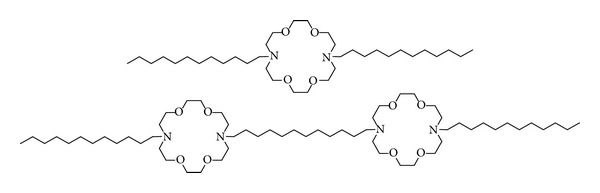
Structural elements of **1** used as controls to study channel function.

The dynamic NMR studies described above established that the synthetic ion channel compounds were effective ion transporters. Numerous control studies were also performed. For example a compound that had two macrocyclic rings connected to each other with a dodecylene chain and terminated by two dodecylene chains was found to be inactive. This molecule was too short to span the bilayer, and it lacked the medial macrocycle. Another control compound was simply *N*,*N *′-didodecyldiaza-18-crown-6. These two “partial channel” elements are pictured in Figure 4. Other structural fragments of compound **1** were studied, but in no case was rapid ion transport detected [33].

### 2.4. Planar Bilayer Conductance

Protein channels [34] normally exhibit four key characteristics. First, they have a pore size dictated by the amino acid sequence that leads to a major conductance state. Second, protein pores ordinarily exhibit rectification. In other words, ions selectively flow in one direction in preference to the other. Third, a protein channel is typically selective for a particular cation, anion, or molecular species. For example a cation channel is selective for positively charged ions and either potassium or sodium or sometimes proton will be favored over other cationic species and certainly over anions. Fourth when the channel is functioning in a bilayer, it typically shows behavior in which the channel undergoes a sequence of open and close events. Typically, when a channel is open, the conductance has a unique magnitude. When the pore is closed, ions fail to flow because the phospholipid bilayer is impermeable. If two channels are simultaneously detected in the open state, the peak observed will be twice the height of that observed for a single channel because the pore size is consistent. Of course, “subconductance states” also occur, but these are beyond the scope of this discussion.

**Figure 5:**
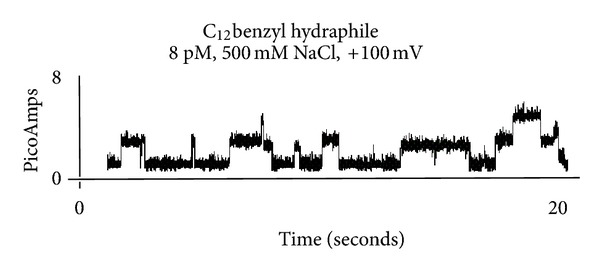
Planar bilayer conductance trace for C_12_ benzyl hydraphile (**4**) ([**4**] = 8 pM), 500 mM aq. NaCl, applied potential = 100 mV.

This so-called “open-close” behavior is detected by an analytical instrument called a planar bilayer conductance voltage clamp apparatus. The term voltage clamp means that the voltage (applied potential) is controlled. A liposome or vesicle has a curved surface, but the membrane present in this device is planar and confined to an orifice of approximately 200 *μ*m. As a result, the instrument is typically called a “bilayer clamp apparatus.” The trace obtained for a synthetic channel-forming compound with a bilayer clamp instrument is shown in Figure 5 along with its structure. This so-called open-close behavior is the signature of channel function. When this pattern is observed for a compound, it confirms that its behavior is mimicking that of protein channels.

### 2.5. Dansyl Channel Fluorescence, Depth Quenching, and Fluorescence Resonance Energy Transfer (FRET)

**Figure 6:**
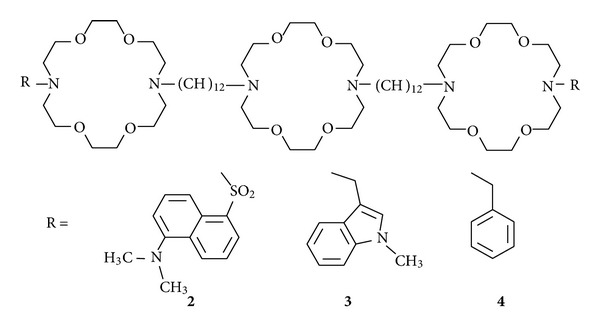


Florescent probes are widely used in the biophysical community [35]. The probes interact directly with other molecules or with the medium to give signals that can be detected at very low concentrations with good accuracy. We prepared a fluorescent synthetic ion channel to use as a probe of structure and activity. The compound is identical in structure to **1** (Figure 1) except that the two terminal dodecyl groups have been replaced by fluorescent dansyl residues (**2**, see Figure 6). The fluorescent maximum observed for dansyl channel **2** depends upon the polarity of the medium in which it is present. We measured *λ*_max⁡_ for the dansyl channel in a range of solvents from nonpolar to polar [36]. We then measured the fluorescence signal for the dansyl channel embedded in a liposomal membrane. The dansyl groups experienced a polarity approximately equal to that of ethanol (*ε* = 20). We inferred from this that the dansyl channel was not globular nor were its head groups in contact with the highly polar aqueous phase (*ε* = 80). 

In another set of experiments, we measured fluorescence resonance energy transfer between the dansyl channel and another channel in which the dansyl groups were replaced by *N*-methylindolyl residues (**3**). Using mixtures of these two channels in molar ratios from 0→1 and 1→0 permitted us to plot the fluorescence response as a function of mole fraction. The slope of this line is reported to correspond to the molecularity of channel function [37]. The slope of the line observed in this experiment was 1.1.

Certain phospholipids are available that have on them a radical quenching group called a tetramethylpyrrolidine *N*-oxide or “Tempo” group. These quenching groups are placed along the fatty acid chain at different distances from the head group. By using several of these phospholipids, it is possible to triangulate the distance between a synthetic ion channel's fluorescent head group, in this case dansyl (**2**), and the midplane of the bilayer [38]. The data obtained in this experiment showed that the dansyl residues were ~15 Å from the midplane of the bilayer. This suggests a separation of the two residues of approximately 30 Å. This also corresponds to the putative thickness of the insulator regime within the bilayer.

Taken together, these data show that the dansyl groups reside in what we call the “midpolar regime.” The midpolar regime is the portion of the phospholipid that contains glycerol and the ester oxygens. The midpolar regime is estimated to have a dielectric constant of 20 to 30 [39]. If the dansyl residues were in contact with water, the fluorescence response would be a larger red shift than was observed experimentally (water *ε* = 80). If the dansyl channel was present in the membrane in a globular conformation, the dansyl groups would exhibit a dielectric constant in the range of 2–5, similar to that within the membrane's insulator regime.

### 2.6. Comparison with the KcsA Channel

A breakthrough in the area of protein channel biochemistry occurred when the structure of the KcsA voltage gated potassium-selective protein channel was reported by Doyle and coworkers in 1998 [40]. So important was this advance that it garnered the Nobel Prize in 2003 [41]. The KcsA channel is a protein, and the conductance pore of which has 8 transmembrane helices. It was described by the authors as having an “inverted tepee” shape. The conductance pore was described as follows. “The overall length of the pore is 45 Å, and its diameter varies along its distance…. From inside the cell (bottom) the pore begins as a tunnel 18 Å in length (the internal pore) and then opens into a wide cavity (~10 Å across) near the middle of the membrane.” This latter description can also be applied to the synthetic ion channel family that we have called the benzyl hydraphiles. 

**Figure 7:**
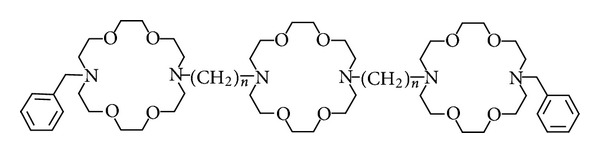
The family of benzyl channels. **5**, *n* = 8; **6**, *n* = 10; **4**, *n* = 12; **7**, *n* = 14; **8**, *n* = 16; **9**, *n* = 18; **10**, *n* = 20.

The hydraphile family is shown in Figure 7 (and above) as **4**–**10**. Compound **4** has 12-carbon spacers, each of which has an extended length of about 15 Å. The N–N distance across a diaza-18-crown-6 unit is about 6 Å. The span from distal nitrogen to distal nitrogen is 48 Å. The benzyl groups extend the molecule farther, and conformational folding would make the overall structure shorter. The twin dodecyl chains comprise the “tunnel” described by MacKinnon and the “cavity…near the middle of the membrane” corresponds to the central macrocycle.

### 2.7. Dansyl Fluorescence in *E. coli*

**Figure 8:**
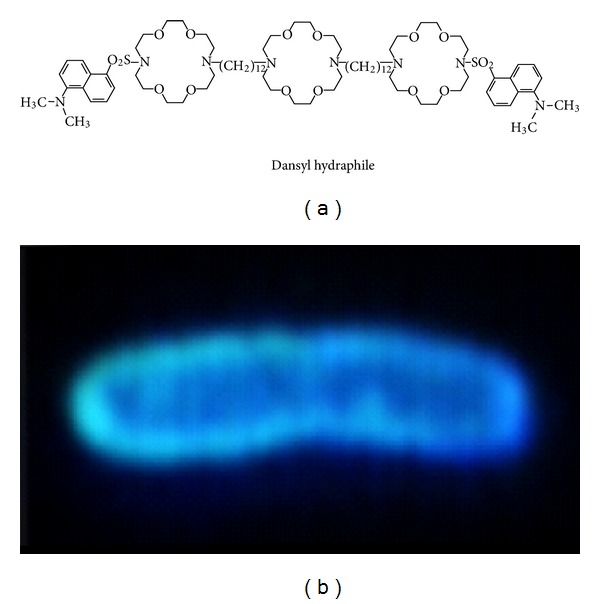
Photomicrograph of *E. coli* exposed to dansyl hydraphile **2**.

To our knowledge, the earliest imaging experiment involving any synthetic ion channel was conducted with a hydraphile having fluorescent side arms. In this particular case, the fluorescent residue was dansyl; the hydraphile is shown above as **2**. It was hypothesized in this experiment that the hydraphile would insert into a cell's plasma membrane and cause the cell's periphery to be fluorescent. The study involved *E. coli* and was conducted using fluorescence microscopy. The images that were obtained showed a fluorescent halo throughout the bacterial periphery, with little infiltration into the cytosol (Figure 8) [16].

The resolution of this fluorescence experiment was not high enough to determine the precise location of the fluorescent residue within the dual external membranes of Gram-negative *E. coli*. Of course, the outer membrane of *E. coli* has channels within it called porins, into which or through which the hydraphile could embed or pass. In the absence of high-resolution data, it appears that the hydraphiles insinuate themselves into both membranes. It is clear that some of the hydraphiles must penetrate both membranes because these synthetic ion channels are toxic to bacteria as well as to yeast at various concentrations. In fact, using a range of hydraphile lengths, we were able to show that the most active channels were most toxic to the bacteria. The inference we drew from this is that the nonrectifying hydraphiles disrupt ion homeostasis. The latter observations are discussed below.

### 2.8. Cellular Toxicity

The toxicity of hydraphiles to bacteria and to yeast was determined initially by using the so-called Kirby-Bauer test [42]. In this assay a petri dish is filled with growth medium and cellulose disks are placed upon the surface. Each of the cellulose disks used was impregnated with a solvent, a control compound, or a compound of interest. Bacteria were then allowed to grow across the surface of the medium. If there is no bacteriostatic effect, the bacteria will grow to the edge of the cellulose disk. If there is a bacteriostatic effect, growth will stop at some distance from the disk. Since the disk is circular, the surrounding dead space is called a “halo.”

**Figure 9:**
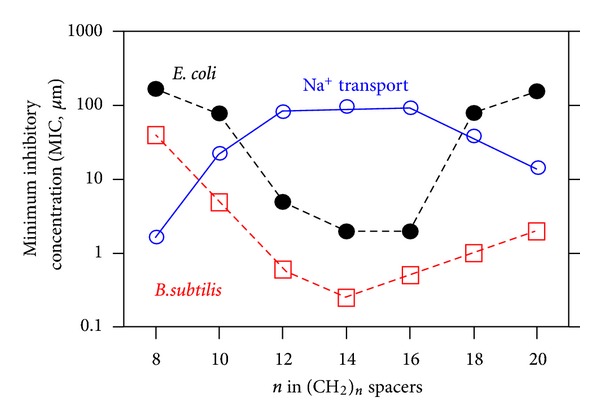
Correspondence between antibacterial activity and chain length of hydraphiles. Sodium transport is recorded as ion release from liposomes on A scale of 0–100%.

When hydraphiles were studied by using this test, toxicity both to bacteria and to yeast was apparent. A hydraphile having relatively short (octylene) spacer chains (**5**) was used as a control. This compound was known to be functionally impaired in bilayer membranes (Figure 9) [43]. Hydraphile **5** has all of the polarity and structural elements that the active hydraphile **4** possesses, but it is too short to span the membrane. Thus, **5** was the ideal control and showed almost no activity in the Kirby-Bauer test. When more quantitative studies were undertaken, it was found that hydraphile **5** (12-carbon spacers) was approximately 15-fold more active than control **5**, which contains 8-carbon spacers. Presumably, the minimal toxicity of **5** resulted from ion carrier activity. 

Detailed studies demonstrated length dependence between hydraphiles and ion transport function in a bilayer membrane [44]. When compounds were too short to span the bilayer, essentially no activity was observed. When compounds were significantly longer than the thickness of a bilayer, function was observed, but it was generally poor. This lower level of activity was attributed to the inability of the longer compounds to achieve an appropriate conductance state conformation. The compounds that had 12- (**4**), 14- (**7**), or 16-carbon (**8**) spacer chains showed good activity and were similar in their ability to conduct sodium ions. The graph of Figure 9 shows this relationship in the upper line labeled “Na^+^ transport.”

The graph of Figure 9 superimposes toxicity results for compounds **4**–**10** with their toxicity either to Gram-negative *E. coli* or to Gram-positive *Bacillus subtilis* [18]. The left ordinate scale shows the minimum inhibitory concentration (MIC) of hydraphile required to kill the bacteria. The superimposed transport of sodium cation is presented on the same logarithmic scale as ion release from liposomes of 0–100%. The plots show a strong inverse relationship, which is especially satisfying considering that chemical and biological responses are being compared. 

### 2.9. Activity of **1** in Human Embryonic Kidney (HEK 293) Cells

Three questions concerning the toxicity of the hydraphiles loomed. First, were they toxic to mammalian cells as well as to prokaryotes and to primary eukaryotes? If so, was there any selectivity among cell types? Third, if the hydraphiles were toxic to mammalian cells, could they still function as ion channels at low (sublethal) concentrations? The last question was answered by using the patch clamp technique on human embryonic kidney (HEK 293) cells [45]. No membrane activity was observed in the absence of added hydraphile **1**. When **1** was added, the electrical activity of the bilayer increased significantly. After a period of observation, the cell was bathed in saline to extract the hydraphile and the electrical activity of the membrane gradually decreased. The cell remained vital throughout the study. Later studies showed that although the hydraphiles were less toxic to mammalian cells than to bacteria or to yeast, the difference was too small to permit their effective use as a therapeutic agent [17]. 

### 2.10. Antibiotic Synergy

Although the selectivity between bacteria and mammalian cells was insufficient to use hydraphiles as medicaments, two possible applications were considered. One of these was to take advantage of the toxicity and use hydraphiles as agents for direct injection chemotherapy. This is discussed in detail in subsequent sections. The other was to use the ability of hydraphiles to penetrate membranes as a synergistic component with known antibiotic compounds. 

**Figure 10:**
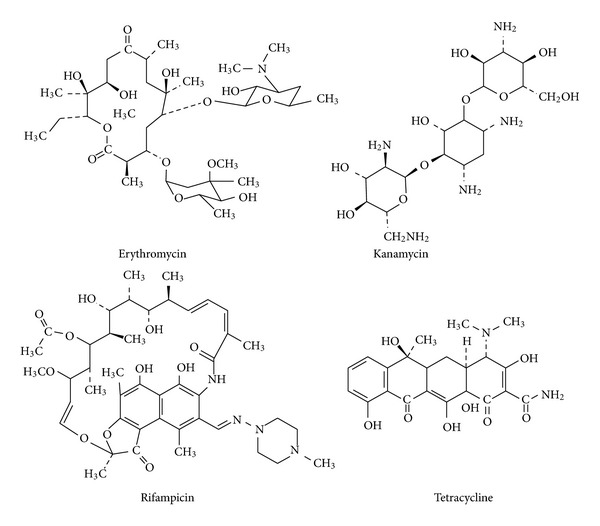


Erythromycin [46], kanamycin [47], rifampicin [48], and tetracycline [49] are all FDA-approved antibiotics (Figure 10). Their structures are very different, although both erythromycin and rifampicin are macrocycles. The mode of action of each of these compounds is known, and each is different from the other. This group of compounds was chosen precisely because of these differences. Experiments were conducted to determine the minimum inhibitory concentrations (MICs) for each antibiotic against several organisms: *E. coli*, *Pseudomonas aeruginosa*, and *B. subtilis*. The toxicity of each antibiotic was then assayed when nontoxic concentrations of hydraphile were present. 

The first significant finding of this study was that the presence of 1 *μ*M **4** or **7** had no effect on the growth of *E. coli*. In the presence of this same concentration of **7**, the activity of the antibiotics studied against *E. coli* was enhanced. When hydraphile **7** was coadministered ([**7**] = 1 *μ*M) with erythromycin, the minimum inhibitory concentration (MIC) observed for the antibiotic changed from 200 *μ*M to 25 *μ*M. This is an 8-fold increase in efficacy or toxic effect. A similar increase was observed for rifampicin: the MIC determined in the absence of hydraphile (50 *μ*M) improved 16-fold to 3.1 *μ*M. Tetracycline is relatively active against *E. coli*; its MIC under the conditions studied was 3.1 *μ*M. In the presence of **7** [1 *μ*M], the MIC decreased (efficacy increased) by 4-fold to 800 nM. 

Additional, unpublished studies have shown that hydraphile synergy applies to other antibiotics and to a variety of prokaryotes or to the yeast *Saccharomyces cerevisiae*. A possible mechanism for the activity is membrane disruption that permits more facile entry of the antibiotic into and through the bilayer. Alternately, the hydraphiles may disrupt the function of efflux pumps that eject the antibiotics. Work is underway to better understand the mechanism(s) by which this synergy occurs. Notwithstanding, higher concentrations of hydraphiles do exhibit cellular toxicity, and this property was used to advantage as discussed in the next section. 

### 2.11. Direct Injection Chemotherapy

A basic principle of chemotherapy is that the rapid growth of tumor cells can be used against them in therapy. Many anticancer drugs are toxins that affect all rapidly growing cells. Thus, tumor cells are susceptible to the poison as are such rapidly growing cells as hair and gut epithelium. If the tumor is localized, it can be surgically excised, although anticancer drugs are often administered as adjuvant or prophylactic agents. 

An alternative to surgery is to directly inject a suitable toxin at the tumor site. The success of such an approach requires that several conditions are met. First, the location of the tumor must be known. Second, the tumor must be localized (not metastasized) as is generally required for a surgical approach. Third, the chemotherapeutic agent must be effective against the tumor. Ideally, the chemical agent will not readily diffuse throughout the adjacent tissue. Both ethanol (EtOH, CH_3_CH_2_OH) and acetic acid (CH_3_COOH) have been studied as direct injection (also called percutaneous injection) therapeutics, and both achieved some success. A key limitation was the fact that these small molecules diffuse rapidly throughout tissue making the desired localization problematic. A potential advantage of hydraphiles in this context is that they insert in bilayers and achieve their toxic effect by disrupting ion homeostasis without rapidly diffusing from the site of injection.

Advantages of the hydraphiles as direct injection chemotherapeutics include their hydrophobicity, their ability to insert in a membrane and disrupt ion homeostasis, and their low proclivity to diffuse within tissue. A range of hydraphiles has been studied, and in all cases, their octanol-water partition coefficients (log⁡⁡P) were at least 10^10^ (i.e., log⁡⁡P > 10) in favor of octanol [15]. The fact that hydraphiles disrupt ion homeostasis was described above. The lack of diffusion was surmised simply by comparing the size and number of polar elements in a hydraphile with a molecule such as ethanol. Of course, experimental confirmation of this property was required. This was done in concert with a study to confirm that rapid tissue damage would take place at the site of hydraphile injection. 

### 2.12. Monitoring Cellular Processes In Vivo

**Figure 11:**
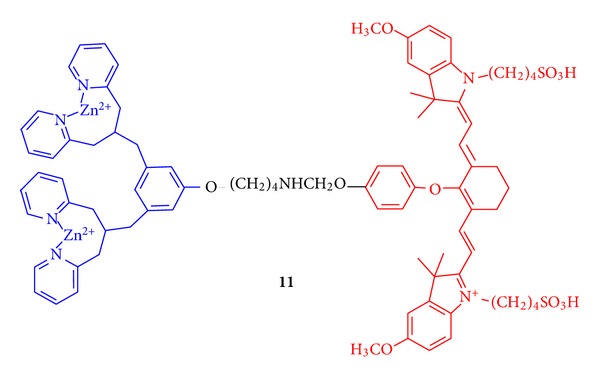


Imaging agents are critical to locating a tumor or disease site and to following the progress of therapy. Compound **11** (Figure 11) was prepared as an imaging probe that could detect anionic phospholipids. It combines a binding site with a near infrared (NIR) fluorescent dye [50]. The two dipicolylamine-zinc(II) cationic binding sites (blue) give the probe an affinity for phosphatidyl serine (PS), and phosphatidyl glycerol (PG). The latter occurs broadly in bacterial membranes and PS is the most abundant anionic lipid that occurs in mammalian cells.

**Figure 12:**
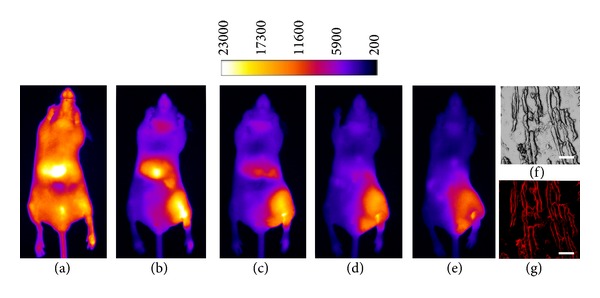
Left: whole animal fluorescence images of probe **11** accumulation at a site of hydraphile muscle damage at 0.1 (A), 3 (B), 6 (C), 12 (D), and 24 (E) h postinjection. Right: histological slice of hydraphile damaged muscle tissue displayed in phase contrast (F) and NIR epifluorescence (G) microscopy modes (scale bars represent 50 *μ*m). Figure reprinted with permission from [51].

The dipicolylamine-zinc(II) affinity group is attached to a carbocyanine fluorophore (red, Cy7) that has an excitation wavelength (*λ*_exc_) at 794 nm and emits at 810 nm. This emission is in the near infrared region and is readily observed from within a living specimen, whereas the emission from conventional fluorescent dyes with green and red wavelengths is obscured by tissue components. A particular advantage of probe **11 **is that apoptosis can be observed because of changes in the internal and external membrane structures that occur upon cell death. Typically, when a mammalian cell dies, phosphatidylserine moves from the internal membrane, where it is abundant, to the extracellular side. The anionic phosphatidylserine head group is complexed by the affinity element of **11**, and the site of cell death can readily be identified by the localization of fluorescence. It has recently been demonstrated that probe **11** will image cell death, as it occurs in mouse muscle tissue treated with various agents including EtOH, ketamine, and compound **4 **(Figure 12, frames A-E). Note that the hydrophobicity of hydraphiles requires that they be dissolved in EtOH, and thus any solvent-related toxicity from it will provide an important baseline for comparison. Of the three agents tested, hydraphile **4 **was the most biologically active with a maximum target/nontarget ratio of 6, compared to a range of 3-4 for ethanol and ketamine [51]. Histology confirmed the muscle tissue damages elicited by **4** and the uptake of NIR probe **11** at the surface of these cells (Figure 12, frames F and G). 

**Figure 13:**
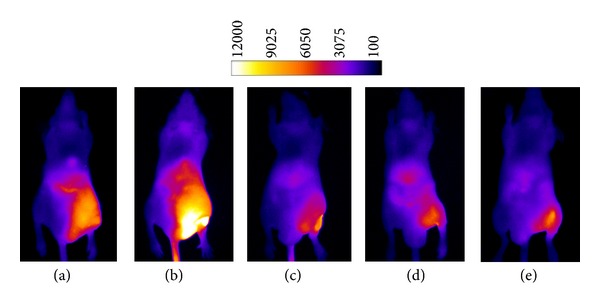
Whole animal fluorescence images of animals with thigh muscle damage induced by **4 **(A), **7** (B), **8** (C), central relay control, **12**, (D), a head group control structure, monobenzyl-diaza-18-crown-6 (E), and 100% EtOH (F). Figure reprinted with permission from [52].

A subsequent in vivo study was conducted to determine the biological activities of an increased number of hydraphile compounds and structural subunits. First, channel length was probed to potentially further enhance muscle tissue damage. The three most active hydraphiles noted during in vitro and antimicrobial studies, **4**, **7**, and **8**, with C_12_, C_14_, and C_16_ chains, were thus tested in vivo using probe **11 **to detect muscle damage. As is apparent in frames A, B, and C of Figure 13, there was no difference between the three compounds, indicating that additional bioactivity could not be gleaned from refinement of channel length. A final in vivo study was conducted to assess the behavior of two structural subunits of **4**. Compound **12** is *N,N *′-didodecyl-4,13-diaza-18-crown-6 and is the central span of the hydraphile, but lacks the two terminal macrocycles. In previous studies in liposomes and mammalian cells, this central relay component showed no tendency to form channels, although it did exhibit toxicity to bacteria and to yeast [52]. Compound **13 **is monobenzyl-4,13-diaza-18-crown-6 and comprises the distal macrocycles that form the channel opening on each side of the membrane. This compound has not demonstrated measurable activity in assays with liposomes, bacteria, or mammalian cells. Panels D and E of Figure 13 show that **12 **and **13 **did not have measurable activity in excess of the EtOH control (frame F) during in vivo studies of tissue damage. These results confirm that hydraphile components themselves will not elicit maximum tissue damage unless they are covalently bound to yield the structure given as **4** [53].

## 3. Conclusions

It has now been just over twenty years since hydraphiles were first conceived and synthesized. Considerable synthetic effort has been dedicated to delineating the structural basis for hydraphile ion transport activity. Further, an exhaustive experimental effort has been maintained over this time period to establish and definitively confirm a channel mechanism for these molecules. This work has evolved from in vitro and biophysical assays to work in pro- and eukaryotic cells and now to living mice. In the latter case, in vivo imaging has been instrumental in establishing that (1) hydraphiles are biologically active toxins that elicit acute tissue damage, (2) structural subunits of hydraphiles will not cause the same level of damage as when covalently bound, and (3) the channel length optimization derived from in vitro and cell studies is also applicable to in vivo studies. The continued evolution of this work provides exciting new opportunities for hydraphiles as therapeutics in a variety of disease models including cancer.
